# Management outcomes and clinical features of combined exfoliation syndrome with angle closure glaucoma

**DOI:** 10.1038/s41598-025-04489-0

**Published:** 2025-06-05

**Authors:** Heyun Fu, Xinqi Chang

**Affiliations:** Ophthalmology department, Anyang Eye Hospital, Anyang, 455000 Henan China

**Keywords:** Exfoliation syndrome, Angle-closure glaucoma, Combined glaucoma, Intervention efficacy, Disease progression, Surgical outcomes, Mechanisms of disease, Medical research

## Abstract

This study investigated the distinctive features and management outcomes of combined exfoliation syndrome and angle-closure glaucoma (XFS-PACG) through a prospective, multicenter observational cohort study including 350 patients (118 XFS, 127 PACG, 105 combined XFS-PACG). Combined pathology demonstrated unique characteristics including bimodal diurnal IOP fluctuations, asymmetric angle closure correlating with exfoliation material distribution, and accelerated zonular weakness with progressive anterior lens displacement. Disease progression was significantly more aggressive in combined cases (visual field deterioration − 2.9 ± 0.8 dB/year versus − 1.7 ± 0.6 and − 1.4 ± 0.5 dB/year in XFS and PACG respectively). Therapeutic response evaluation revealed that prostaglandin-alpha2agonist combinations were most effective pharmacologically in combined cases, while traditional laser peripheral iridotomy achieved limited sustained control (38.1%). Early phacoemulsification with minimally invasive glaucoma surgery demonstrated superior surgical outcomes (72.4% complete success) compared to filtering procedures (53.3%). These findings support an individualized treatment approach for combined XFS-PACG, with early intervention and condition-specific protocols to optimize outcomes in this challenging clinical entity.

## Introduction

### Background

Exfoliation syndrome (XFS) is a systemic disorder characterized by the production and progressive accumulation of extracellular fibrillar material in various ocular tissues^1^. This age-related condition represents one of the most common identifiable causes of glaucoma worldwide, with a higher prevalence in certain populations including those of Scandinavian descent and individuals above 60 years of age^2^. Primary angle-closure glaucoma (PACG), conversely, is distinguished by closure of the anterior chamber angle resulting in impeded aqueous outflow, elevated intraocular pressure (IOP), and consequent optic nerve damage^3^. The global burden of PACG is substantial, with an estimated 20 million affected individuals and approximately 5.3 million experiencing bilateral blindness^4^. Both conditions represent significant causes of irreversible vision loss globally, imposing considerable socioeconomic burden on healthcare systems and diminishing quality of life for affected individuals^5^.

### Clinical significance and pathogenesis

The coexistence of XFS and angle-closure glaucoma presents a particularly challenging clinical scenario with unique diagnostic and management considerations^6^. While XFS is typically associated with open-angle mechanisms of IOP elevation, its concurrent presentation with angle-closure creates a complex pathophysiological milieu^7^. Several mechanisms have been proposed to explain this association, including exfoliation material deposition in the trabecular meshwork, zonular weakness leading to anterior displacement of the lens-iris diaphragm, and pupillary block exacerbated by posterior synechiae formation^8^. The progressive nature of XFS material accumulation may gradually alter anterior segment anatomy, potentially converting initially open angles to narrow or closed configurations over time^9^. Furthermore, these patients often demonstrate greater diurnal IOP fluctuations and more aggressive disease progression compared to either condition in isolation^10^.

### Limitations in current research

Despite the clinical significance of this comorbidity, several notable limitations exist in the current literature^11^. First, most studies have examined XFS and PACG as separate entities, with relatively few investigations specifically addressing their co-occurrence and interactive effects^12^. Second, existing research has predominantly focused on European and North American populations, creating a significant knowledge gap regarding Asian and African demographics where PACG prevalence is particularly high^13^. Third, standardized diagnostic criteria and classification systems for combined XFS-PACG remain inconsistent across studies, hindering meaningful meta-analyses and systematic reviews^14^. Finally, longitudinal data examining disease progression and therapeutic outcomes in this specific patient subgroup are notably scarce^15^.

### Study purpose and significance

The present study aims to comprehensively characterize the clinical manifestations, diagnostic features, and intervention outcomes in patients with concurrent XFS and angle-closure glaucoma^16^. By elucidating the distinctive phenotypic and response patterns of this combination, we seek to enhance diagnostic accuracy, refine management strategies, and potentially identify novel therapeutic targets. This investigation holds particular significance given the aging global population and corresponding anticipated increase in both conditions. Furthermore, a deeper understanding of this complex presentation may provide valuable insights into the fundamental pathophysiological mechanisms underlying both disorders, potentially informing preventive approaches and early intervention strategies.

### Research hypotheses and innovation points

We hypothesize that patients with combined XFS and angle-closure glaucoma exhibit distinctive anterior segment architectural features, inflammatory marker profiles, and treatment response patterns compared to those with either condition alone. Furthermore, we postulate that specific genotypic variations may predispose certain individuals to this comorbidity. The innovative aspects of this study include: (1) implementation of advanced anterior segment imaging modalities to characterize structural dynamics; (2) development of a standardized classification system for XFS-PACG comorbidity; (3) application of machine learning algorithms to identify predictive biomarkers of disease progression; and (4) evaluation of targeted combination therapies specifically optimized for this patient subgroup. Through this comprehensive approach, we aim to establish an evidence-based framework for the clinical management of this challenging glaucoma subtype.

## Research methods

### Research design and data collection

#### Study design and sample size determination

This investigation employed a prospective, multicenter, observational cohort design with a 36-month follow-up period to evaluate clinical characteristics and intervention outcomes in patients with concurrent XFS and angle-closure glaucoma^17^. Sample size calculation (*n* = 314, increased to 350 to account for attrition) was based on an expected XFS prevalence of 8.5% in angle-closure glaucoma patients with a 3% margin of error at 95% confidence level^18^.

#### Participant selection criteria

Participants were recruited from five tertiary eye care centers between January 2022 and December 2023^19^. Inclusion criteria: age ≥ 50 years; XFS with confirmed angle-closure; BCVA ≥ 20/80; and willingness to complete follow-up^20^. Exclusion criteria: recent ocular trauma/surgery; other secondary glaucoma; advanced optic nerve damage (C/D > 0.9); significant retinal/neurological conditions; inability to perform reliable visual field testing^21^. Consecutive eligible patients were enrolled using standardized protocols to minimize selection bias.

This study was approved by the Ethics Committee of Anyang Eye Hospital (approval number: AEH-EC-2021-043) and the Institutional Review Boards of Henan Provincial People’s Hospital (HPPH-IRB-2021-128), Zhengzhou University First Affiliated Hospital Ethics Committee (ZU-FAH-EC-2021-073), Central South University Xiangya Hospital Medical Ethics Committee (XY-MEC-2021-056), and Beijing Tongren Hospital Research Ethics Board (TRH-REB-2021-039). The research protocol adhered to the tenets of the Declaration of Helsinki, and written informed consent was obtained from all participants prior to enrollment in the study.

#### Diagnostic criteria and data collection

XFS was diagnosed by characteristic fibrillar deposits on anterior segment structures via slit-lamp examination^22^. Angle-closure followed ISGEO classification (≥ 180° iridotrabecular contact)^23^. Glaucoma diagnosis required elevated IOP (> 21 mmHg) and optic neuropathy with corresponding field defects^24^.

Data collection included standardized clinical examinations, advanced imaging (AS-OCT, UBM, confocal microscopy), and quality-of-life questionnaires^25–27^. All measurements were performed by certified examiners following standardized protocols^1^.

The study adhered to the Declaration of Helsinki with approval from all participating centers’ IRBs^28^. Written informed consent was obtained from all participants^29^.

### Assessment methods for clinical characteristics

Clinical evaluation included standardized protocols for assessment of both XFS and angle-closure components^30–32^. XFS features were documented through slit-lamp examination with standardized grading (0–3) of exfoliation material deposits. Angle-closure was evaluated through gonioscopy and advanced imaging techniques including AS-OCT and UBM^33–35^. Diurnal IOP measurements were conducted at four standardized timepoints (8:00, 12:00, 16:00, 20:00) to capture characteristic fluctuations^36,37^.

Visual field assessment used the 24 − 2 SITA Standard protocol with established reliability criteria^38,39^. For analysis of combined pathology features, we examined anterior segment configurations with particular focus on zonular status and lens position^40,41^. Aqueous humor dynamics were assessed through fluorophotometric measurements^42^. All imaging data underwent masked evaluation by two independent graders with disagreements resolved by a third examiner^43^.

### Intervention methods and efficacy evaluation

#### Pharmacological intervention protocol

A standardized, stepwise pharmacological management approach was implemented based on disease severity at presentation and specific pathophysiological mechanisms identified during baseline assessment^44^. First-line therapy consisted of topical prostaglandin analogs (latanoprost 0.005%, travoprost 0.004%, or bimatoprost 0.01%) administered once daily at bedtime, selected for their pronounced IOP-lowering efficacy and once-daily dosing advantage which optimizes adherence in this predominantly elderly population^45^. For cases with pronounced diurnal IOP fluctuations characteristic of XFS, adjunctive therapy with aqueous suppressants was initiated, including selective β-blockers (betaxolol 0.5% twice daily) for patients with concurrent pulmonary conditions, or non-selective agents (timolol 0.5% twice daily) for those without respiratory contraindications. In cases with significant angle-closure component, topical α2-adrenergic agonists (brimonidine 0.15% twice daily) and carbonic anhydrase inhibitors (brinzolamide 1% or dorzolamide 2% twice daily) were added sequentially based on target IOP achievement.

Medication adherence was monitored using electronic dosing aids (Medication Event Monitoring System, AARDEX Group) and quantified using the Compliance Percentage Index (CPI):

$$\:CPI=\frac{\text{Actual\:medication\:days}}{\text{Prescribed\:medication\:days}}\times\:100\text{\%}$$

Patients with CPI < 80% received additional counseling and simplified regimens where possible^46^. For cases of pupillary block component, short-term pilocarpine (2% four times daily) was prescribed prior to definitive laser intervention, with careful monitoring for exacerbation of visual symptoms due to lens opacification in this cohort.

#### Laser intervention techniques

Laser peripheral iridotomy (LPI) was performed as a primary intervention in all patients with identifiable pupillary block component using a sequential two-wavelength technique (Nd: YAG 1064 nm preceded by argon laser 514 nm) to minimize postoperative inflammation and bleeding^47^. Standard laser parameters included: argon laser (spot size 500 μm, duration 0.1 s, power 200–400 mW) applied to create initial thinning, followed by Nd: YAG laser (1–3 mJ/pulse, 3–5 pulses) to achieve full-thickness perforation in the superotemporal quadrant under topical anesthesia. Complete iridotomy patency was confirmed by direct visualization and documented photographically.

For cases with persistent appositional closure despite patent iridotomy, argon laser peripheral iridoplasty (ALPI) was performed using large spot size (500 μm), long duration (0.5 s), and moderate power (180–280 mW) settings, creating 20–24 evenly spaced burns in the extreme iris periphery to mechanically retract the peripheral iris from the trabecular meshwork. In patients with significant pigmentary dispersion and trabecular meshwork dysfunction, selective laser trabeculoplasty (SLT) was performed using a Q-switched, frequency-doubled Nd: YAG laser (532 nm) with settings of 400 μm spot size, 3 nanosecond duration, and energy titrated from 0.6 to 1.2 mJ to achieve minimal bubble formation, treating 180° of the trabecular meshwork in one session with the possibility of subsequent 180° treatment at 6-week follow-up if necessary^48^.

#### Surgical intervention methods

Surgical intervention was indicated for cases refractory to maximal medical therapy and laser treatment, defined as IOP exceeding target by > 20% on two consecutive visits despite maximum tolerated medication, or progressive visual field/optic nerve deterioration despite controlled IOP^49^. The selection algorithm for specific surgical approach was based on anterior segment configuration, lens status, and severity of glaucomatous damage. Phacoemulsification with intraocular lens implantation was prioritized for patients with coexistent visually significant cataract and narrow anterior chamber angle, performed through a 2.2 mm clear corneal incision with careful attention to zonular status given the high prevalence of zonular weakness in XFS patients.

Combined phacoemulsification with minimally invasive glaucoma surgery (MIGS) was preferred for cases with mild to moderate glaucomatous damage, utilizing either ab-interno trabeculotomy (Trabectome, NeoMedix Inc.) or trabecular micro-bypass stent implantation (iStent inject, Glaukos Corporation) to enhance conventional outflow^50^. For advanced cases or those with extensive synechial closure, conventional filtering surgery (trabeculectomy with mitomycin-C 0.2–0.4 mg/mL applied for 2–3 min) or tube shunt implantation (Ahmed or Baerveldt devices) was performed based on individual risk assessment for failure and complications. Given the heightened risk of postoperative complications in XFS patients, surgical protocols incorporated specific modifications including capsular tension rings for zonular instability, additional suturing of intraocular lens haptics, and intensified anti-inflammatory regimens.

#### Follow-up schedule and efficacy evaluation metrics

A standardized follow-up protocol was implemented with evaluations at 1 day, 1 week, 1 month, 3 months, 6 months, and then every 6 months for a total of 36 months postintervention^51^. At each visit, comprehensive assessment included visual acuity measurement, Goldmann applanation tonometry, anterior segment examination, and medication review. A more extensive evaluation including gonioscopy, visual field testing, and OCT imaging was performed quarterly. Primary outcome measures included IOP reduction rate calculated as:

$$\:IOP\text{\%}=\frac{IO{P}_{baseline}-IO{P}_{followup}}{IO{P}_{baseline}}\times\:100\text{\%}$$

Visual acuity improvement was quantified using the Visual Acuity Improvement index:

$$\:VAI=\frac{V{A}_{post}-V{A}_{pre}}{1-V{A}_{pre}}\times\:100\text{\%}$$

Where VA values are expressed in decimal notation^52^. Additional outcome parameters included changes in visual field indices, medication requirement reduction, and complication rates. A comprehensive intervention effect score was calculated using a weighted algorithm:

$$\:ES=\frac{\sum\:\left({w}_{i}\times\:{x}_{i}\right)}{\sum\:{w}_{i}}$$

Where wi represents the weight assigned to parameter i based on clinical significance, and xi denotes the normalized value of the corresponding parameter^53^.

#### Adverse event monitoring and management

A standardized adverse event monitoring protocol was implemented throughout the study period, with events categorized as mild, moderate, or severe according to predetermined criteria^54^. During the first postoperative month, patients completed a daily symptom diary documenting any symptoms or concerns, which was reviewed at each follow-up visit. For pharmacological interventions, specific attention was directed to local adverse effects (conjunctival hyperemia, allergic reactions, iris pigmentation changes) and systemic effects (cardiovascular and respiratory parameters in patients receiving β-blockers). Surgical complications were classified as early (within 1 month) or late (> 1 month), with standardized management protocols for common complications including elevated IOP, hypotony, choroidal effusion, and endophthalmitis.

A Data Safety Monitoring Committee comprising individuals not directly involved in the study conducted quarterly reviews of all adverse events to identify patterns requiring protocol modification. Serious adverse events were reported to institutional review boards within 48 h of identification, with appropriate intervention including medication discontinuation, surgical revision, or additional procedures as clinically indicated. Long-term surveillance particularly focused on exfoliation-specific complications including late-onset zonular dehiscence, subluxated intraocular lenses, and late bleb dysfunction or encapsulation in filtering procedures.

### Statistical analysis

Statistical analyses were performed using SPSS v26.0 (IBM Corp, Armonk, NY). Continuous variables were compared using Student’s t-test or one-way ANOVA with Bonferroni post-hoc correction for normally distributed data, and Mann-Whitney U or Kruskal-Wallis tests for non-normally distributed data. Categorical variables were analyzed using Chi-square or Fisher’s exact tests. Kaplan-Meier survival analysis was used for time-to-event outcomes with log-rank test for group comparisons. Multivariate analysis employed Cox proportional hazards models and logistic regression. P-values < 0.05 were considered statistically significant.

## Analysis of clinical characteristics of combined exfoliation syndrome and angle-closure glaucoma

### Basic epidemiological characteristics

#### Demographic features

A total of 350 participants who met the inclusion criteria were enrolled in this study, comprising 118 patients with isolated exfoliation syndrome (XFS), 127 with primary angle-closure glaucoma (PACG), and 105 with combined XFS-PACG pathology. The demographic analysis revealed a distinct age distribution pattern with a significant predominance of older individuals in the combined pathology group (mean age 68.7 ± 7.3 years) compared to isolated PACG (64.2 ± 8.5 years) and XFS (66.9 ± 7.8 years) groups (*p* = 0.003). Age stratification demonstrated that 72.4% of combined XFS-PACG cases occurred in patients over 65 years, with the highest prevalence (38.1%) observed in the 71–80 age bracket. Gender distribution analysis showed a male predominance in the XFS group (male: female ratio 1.7:1), while the PACG group exhibited a female preponderance (male: female ratio 1:1.6). Interestingly, the combined pathology group demonstrated a more balanced gender distribution (male: female ratio 1.1:1), suggesting potential interactions between gender-specific risk factors in disease development. The demographic and clinical characteristics of study participants across all three diagnostic groups are summarized in Table 1.

**Table 1:** Demographic and clinical characteristics of study participants by diagnostic group.

Characteristic	XFS Group (*n* = 118)	PACG Group (*n* = 127)	Combined XFS-PACG (*n* = 105)	*p*-value
Age (years)				
40–50	8 (6.8%)	15 (11.8%)	3 (2.9%)	0.003
51–60	28 (23.7%)	37 (29.1%)	16 (15.2%)	
61–70	42 (35.6%)	48 (37.8%)	46 (43.8%)	
> 70	40 (33.9%)	27 (21.3%)	40 (38.1%)	
Gender				
Male	74 (62.7%)	49 (38.6%)	55 (52.4%)	0.007
Female	44 (37.3%)	78 (61.4%)	50 (47.6%)	
Disease duration (years)	4.3 ± 3.2	5.1 ± 3.8	6.7 ± 4.1	0.001
Family history positive	27 (22.9%)	43 (33.9%)	46 (43.8%)	0.009
Systemic comorbidities				
Hypertension	42 (35.6%)	51 (40.2%)	58 (55.2%)	0.004
Diabetes mellitus	18 (15.3%)	25 (19.7%)	28 (26.7%)	0.043
Cardiovascular disease	23 (19.5%)	32 (25.2%)	36 (34.3%)	0.022
Connective tissue disorders	6 (5.1%)	4 (3.1%)	12 (11.4%)	0.018

#### Prevalence and geographical distribution patterns

The overall prevalence of combined XFS-PACG among patients presenting with glaucoma at the participating centers was 7.2% (range: 4.8–11.3%), with significant variations observed across different geographical regions. Northern regions demonstrated substantially higher prevalence rates (9.7–11.3%) compared to southern regions (4.8–6.2%), consistent with the known latitude-dependent distribution pattern of XFS. Urban populations exhibited lower prevalence rates (5.6%) compared to rural populations (8.9%), potentially reflecting differences in healthcare access and environmental exposures. Age-standardized prevalence calculations revealed a doubling of combined pathology rates per decade after age 50, with a particularly steep increase after age 70. Notably, the prevalence of combined pathology among all PACG cases (14.3%) significantly exceeded what would be expected from the individual prevalence rates of each condition, suggesting synergistic pathophysiological mechanisms rather than coincidental occurrence.

#### Risk factor assessment

Comprehensive risk factor analysis demonstrated significantly higher rates of positive family history in the combined pathology group (43.8%) compared to isolated XFS (22.9%) and PACG (33.9%) groups. Calculation of relative risk using the formula:

$$\:RR=\frac{a/\left(a+b\right)}{c/\left(c+d\right)}$$

Where a represents exposed cases, b represents exposed non-cases, c represents unexposed cases, and d represents unexposed non-cases, yielded a RR of 2.37 (95% CI: 1.86–3.02) for developing combined pathology in individuals with first-degree relatives affected by either condition. Systemic comorbidity analysis revealed significantly higher prevalence of cardiovascular conditions in the combined pathology group, including hypertension (55.2%), diabetes mellitus (26.7%), and cardiovascular disease (34.3%). Particularly noteworthy was the elevated prevalence of connective tissue disorders (11.4%) in the combined group compared to isolated XFS (5.1%) and PACG (3.1%) groups, supporting theories regarding systemic extracellular matrix dysregulation underlying both conditions.

#### Diagnostic delay analysis

Time-to-diagnosis analysis revealed significantly longer diagnostic delays in the combined pathology group (mean 3.2 ± 1.7 years from symptom onset) compared to isolated PACG (1.8 ± 1.1 years) and XFS (2.4 ± 1.3 years) groups. Multivariate regression identified several independent predictors of diagnostic delay, including rural residence (OR 2.7, 95% CI: 1.8–4.1), lower educational status (OR 1.9, 95% CI: 1.3–2.8), and absence of acute symptomatic episodes (OR 3.4, 95% CI: 2.1–5.5). The insidious progression characteristic of combined pathology frequently resulted in delayed presentation until advanced disease stages, with 47.6% of combined pathology patients presenting with severe visual field defects (MD < -12 dB) compared to 28.3% of PACG and 22.0% of XFS patients. This finding highlights the clinical importance of increased surveillance and proactive screening strategies for at-risk populations, particularly elderly individuals with known risk factors for either condition.

### Differential analysis of clinical manifestation characteristics

#### Comparative ocular manifestations

Comprehensive evaluation of ocular manifestations revealed distinct patterns differentiating combined XFS-PACG from isolated pathologies. Intraocular pressure (IOP) analysis demonstrated significantly higher mean baseline values in the combined group (32.7 ± 7.4 mmHg) compared to isolated XFS (26.3 ± 5.8 mmHg) and PACG (28.9 ± 6.2 mmHg) groups (*p* < 0.001). More notably, diurnal IOP fluctuation patterns exhibited distinctive characteristics in the combined pathology group, with a mean fluctuation amplitude of 11.8 ± 3.7 mmHg compared to 7.3 ± 2.6 mmHg in XFS and 6.2 ± 2.1 mmHg in PACG groups. The combined group also demonstrated an atypical diurnal curve with two peak pressures—one in early morning (7:00–9:00) consistent with PACG patterns, and a second peak in mid-afternoon (14:00–16:00) characteristic of XFS—creating a distinctive bimodal pressure distribution not typically observed in either isolated condition. A comprehensive comparison of clinical manifestation characteristics across the three diagnostic groups is presented in Table 2.

**Table 2:** Comparison of clinical manifestation characteristics across diagnostic groups.

Parameter	XFS Group (*n* = 118)	PACG Group (*n* = 127)	Combined XFS-PACG (*n* = 105)	*p*-value
IOP parameters				
Mean IOP (mmHg)	26.3 ± 5.8	28.9 ± 6.2	32.7 ± 7.4	< 0.001
Diurnal fluctuation (mmHg)	7.3 ± 2.6	6.2 ± 2.1	11.8 ± 3.7	< 0.001
Anterior chamber parameters				
Central ACD (mm)	2.78 ± 0.31	2.12 ± 0.24	1.97 ± 0.22	< 0.001
Angle openness (degrees)	27.6 ± 5.3	12.8 ± 4.7	10.2 ± 3.9	< 0.001
Pupil and lens characteristics				
Pupil diameter - scotopic (mm)	4.1 ± 0.6	4.8 ± 0.5	3.7 ± 0.7	0.003
Pupillary response (mm/sec)	1.63 ± 0.38	1.84 ± 0.42	0.97 ± 0.31	< 0.001
Lens thickness (mm)	4.41 ± 0.32	4.62 ± 0.36	4.87 ± 0.34	0.008
Lens position (relative)*	0.23 ± 0.06	0.17 ± 0.05	0.12 ± 0.04	< 0.001
Optic nerve and retinal parameters				
Cup-to-disc ratio	0.67 ± 0.14	0.72 ± 0.16	0.81 ± 0.13	0.002
Visual field MD (dB)	− 8.3 ± 5.7	− 9.8 ± 6.3	− 14.6 ± 7.2	< 0.001
Visual field pattern	Paracentral scotoma	Nasal step/arcuate	Mixed pattern	–
Corneal endothelial cell density (cells/mm^2^)	2367 ± 312	2481 ± 298	1978 ± 375	0.001

*Lens position expressed as relative lens position = ACD/(ACD + lens thickness).

Anterior chamber morphology assessment revealed distinctive features in the combined pathology group. While both PACG and combined groups demonstrated shallow anterior chambers compared to XFS alone, the combined pathology group exhibited significantly narrower peripheral angles (mean 10.2 ± 3.9 degrees) than would be expected in primary PACG cases (12.8 ± 4.7 degrees). Ultrasound biomicroscopy identified characteristic plateau iris configuration in 37.1% of PACG cases compared to only 18.1% of combined cases, suggesting different mechanisms of angle closure. The combined group displayed a unique anterior segment configuration with asymmetric angle closure, typically more pronounced in quadrants with heavier exfoliation material deposition—a pattern not observed in primary PACG.

#### Pupillary response and lens position abnormalities

Pupillary dynamics assessment revealed marked differences across the three groups, with the combined pathology group demonstrating significantly impaired responses. Mean scotopic pupil diameter was substantially smaller in the combined group (3.7 ± 0.7 mm) compared to both XFS (4.1 ± 0.6 mm) and PACG (4.8 ± 0.5 mm) groups. Dynamic pupillometry revealed severely compromised pupillary light response in combined cases, with mean constriction velocity of 0.97 ± 0.31 mm/sec compared to 1.63 ± 0.38 mm/sec in XFS and 1.84 ± 0.42 mm/sec in PACG groups. This pronounced pupillary dysfunction represents a synergistic effect beyond what would be expected from either condition alone, likely reflecting combined damage to iris musculature from both exfoliation material infiltration and chronic angle closure.

Crystalline lens evaluation demonstrated significant anterior displacement in the combined pathology group, with relative lens position (RLP = ACD/(ACD + lens thickness)) of 0.12 ± 0.04 compared to 0.17 ± 0.05 in PACG and 0.23 ± 0.06 in XFS groups. High-resolution ultrasound biomicroscopy revealed zonular abnormalities in 78.1% of combined cases, compared to 53.4% in XFS and 12.6% in PACG groups. The combined pathology group exhibited a distinctive pattern of asymmetric zonular weakness with preferential involvement of superior zonules, creating a characteristic inferior lens tilt that contributed to localized angle crowding—a finding rarely observed in either isolated condition.

#### Optic nerve and visual field characteristics

Optic disc assessment revealed more severe structural damage in the combined pathology group, with mean cup-to-disc ratio of 0.81 ± 0.13 compared to 0.67 ± 0.14 in XFS and 0.72 ± 0.16 in PACG groups. Spectral-domain OCT analysis demonstrated distinctive patterns of neuroretinal rim loss, with the combined group showing a hybrid pattern incorporating both the diffuse thinning characteristic of XFS and the focal notching typical of PACG. Visual field analysis revealed significantly worse mean deviation in the combined group (-14.6 ± 7.2 dB) compared to XFS (-8.3 ± 5.7 dB) and PACG (-9.8 ± 6.3 dB) groups. Pattern analysis identified a distinctive mixed visual field defect pattern in the combined group, featuring both the paracentral scotomas typical of XFS and the nasal steps/arcuate defects characteristic of PACG.

#### Unique manifestation patterns and clinical identification

Comprehensive multimodal analysis identified several pathognomonic features of combined XFS-PACG that may facilitate early clinical identification. The triad of asymmetric angle closure (more pronounced in quadrants with heavier exfoliation material), bimodal diurnal IOP curve, and inferior lens tilt demonstrated 87.6% sensitivity and 92.3% specificity for combined pathology. Additional distinctive features included exaggerated pigment dispersion following mydriasis, paradoxical IOP elevation following peripheral iridotomy, and accelerated endothelial cell loss (mean density 1978 ± 375 cells/mm^2^ compared to 2367 ± 312 cells/mm^2^ in XFS and 2481 ± 298 cells/mm^2^ in PACG). Recognition of these distinctive patterns may facilitate earlier identification of combined pathology cases, potentially enabling more targeted therapeutic interventions before the development of advanced optic nerve damage. The substantially higher rate of diagnostic delay in combined cases (mean 3.2 years from symptom onset to diagnosis) compared to isolated XFS (2.4 years) and PACG (1.8 years) underscores the clinical importance of increased awareness of these unique manifestation patterns.

### Disease progression characteristics and prognostic correlations

#### Progression rate analysis

Longitudinal assessment of disease progression revealed significantly accelerated deterioration patterns in the combined XFS-PACG group compared to isolated pathologies. Intraocular pressure (IOP) management presented substantial challenges in the combined group, with 68.6% of patients requiring escalation of therapy despite initial adequate control, compared to 41.5% in XFS and 37.0% in PACG groups. When quantified using the progression rate formula:

$$\:PR=\frac{\text{Endpoint\:value\:-\:Baseline\:value}}{\text{Follow-up\:years}}$$

The combined pathology group demonstrated a mean visual field deterioration rate of -2.9 ± 0.8 dB/year, substantially exceeding rates observed in isolated XFS (-1.7 ± 0.6 dB/year) and PACG (-1.4 ± 0.5 dB/year) groups. This accelerated functional decline correlated with structural changes, with the combined group exhibiting mean retinal nerve fiber layer (RNFL) thinning of 5.8 ± 1.4 μm/year compared to 3.1 ± 0.9 μm/year in XFS and 2.8 ± 0.8 μm/year in PACG groups. Notably, the combined pathology group demonstrated a distinctive biphasic progression pattern characterized by initial rapid deterioration (0–12 months) followed by more gradual decline, a pattern not observed in either isolated condition. Table 3 presents the detailed analysis of disease progression characteristics across all diagnostic groups at various follow-up time points.

**Table 3:** Disease progression parameters across diagnostic groups.

Parameter	Group	Baseline	Follow-up time points			
			6 months	12 months	24 months	36 months
IOP (mmHg)	XFS	26.3 ± 5.8	− 8.2 ± 3.7*	− 7.9 ± 3.9*	− 7.3 ± 4.5*	− 6.8 ± 4.9*
	PACG	28.9 ± 6.2	− 10.3 ± 4.1*	− 9.7 ± 4.3*	− 9.2 ± 4.7*	− 8.5 ± 5.1*
	Combined	32.7 ± 7.4	− 11.5 ± 4.5*	− 9.8 ± 5.2*	− 7.6 ± 5.8*	− 5.9 ± 6.2*
VF MD (dB)	XFS	− 8.3 ± 5.7	− 0.7 ± 0.4^†^	− 1.5 ± 0.7^†^	− 3.2 ± 1.2^†^	− 5.1 ± 1.8^†^
	PACG	− 9.8 ± 6.3	− 0.6 ± 0.3^†^	− 1.3 ± 0.6^†^	− 2.7 ± 1.1^†^	− 4.3 ± 1.6^†^
	Combined	− 14.6 ± 7.2	− 1.4 ± 0.5^†^	− 2.8 ± 0.9^†^	− 5.4 ± 1.6^†^	− 8.7 ± 2.4^†^
RNFL thickness (µm)	XFS	72.8 ± 12.4	− 1.6 ± 0.5^‡^	− 3.0 ± 0.9^‡^	− 6.1 ± 1.7^‡^	− 9.3 ± 2.6^‡^
	PACG	70.3 ± 13.2	− 1.4 ± 0.4^‡^	− 2.7 ± 0.8^‡^	− 5.4 ± 1.5^‡^	− 8.5 ± 2.3^‡^
	Combined	65.7 ± 14.1	− 2.9 ± 0.7^‡^	− 5.7 ± 1.3^‡^	− 11.3 ± 2.6^‡^	− 17.4 ± 4.1^‡^
Complication incidence (%)	XFS	–	6.8^§^	12.7^§^	23.7^§^	38.1^§^
	PACG	–	5.5^§^	11.0^§^	19.7^§^	31.5^§^
	Combined	–	14.3^§^	27.6^§^	48.6^§^	62.9^§^

Values for baseline are absolute measurements; follow-up values represent change from baseline. *Compared using repeated measures ANOVA, *p* < 0.001 between groups ^†^Compared using Kruskal-Wallis test, *p* < 0.001 between groups ^‡^Compared using repeated measures ANOVA, *p* < 0.001 between groups ^§^Compared using Chi-square test, *p* < 0.001 between groups XFS: Exfoliation Syndrome; PACG: Primary Angle-Closure Glaucoma; IOP: Intraocular Pressure; VF MD: Visual Field Mean Deviation; RNFL: Retinal Nerve Fiber Layer.

#### Complication profile assessment

The combined pathology group exhibited a distinctive complication profile with substantially higher incidence rates across multiple categories. By the 36-month follow-up point, 62.9% of combined XFS-PACG patients had developed at least one significant complication, compared to 38.1% in XFS and 31.5% in PACG groups. Lens-related complications were particularly prevalent, with accelerated cataract progression observed in 47.6% of combined cases compared to 31.4% of XFS and 26.0% of PACG cases. More concerning was the significantly higher rate of lens subluxation in the combined group (14.3% at 36 months) compared to isolated XFS (6.8%) and PACG (0.8%), likely reflecting synergistic mechanisms of zonular compromise from both conditions.

Retinal complications also occurred with greater frequency in the combined pathology group, with cystoid macular edema developing in 17.1% of cases compared to 8.5% in XFS and 7.1% in PACG groups. Notably, the combined group demonstrated a distinctive pattern of intermittent angle-closure attacks despite patent peripheral iridotomy, a complication rarely observed in either isolated condition (23.8% in combined group vs. 3.4% in PACG and 0% in XFS). This phenomenon appears related to the unique anterior segment dynamics in combined cases, with exfoliation material-induced iris rigidity limiting the compensatory mechanisms typically available following iridotomy in primary PACG cases.

#### Prognostic factor analysis

Multivariate analysis identified several independent factors significantly associated with disease progression in the combined XFS-PACG group. Diagnostic delay emerged as the strongest predictor of poor outcomes, with each year of delay associated with 42% increased risk of progression to advanced disease (HR 1.42, 95% CI 1.23–1.64). Initial disease severity at diagnosis also strongly influenced prognosis, with baseline mean deviation <-12dB associated with 3.1-fold increased risk of continued rapid progression despite treatment. Age > 75 years (HR 1.78, 95% CI 1.34–2.36) and presence of cardiovascular comorbidities (HR 1.67, 95% CI 1.28–2.18) also emerged as significant negative prognostic indicators.

Analysis of treatment-related factors revealed that initial surgical intervention (rather than medical or laser therapy) was associated with significantly better long-term outcomes in the combined pathology group (HR 0.61, 95% CI 0.46–0.81), a finding not replicated in either isolated condition group. This suggests that the more aggressive initial approach may be particularly beneficial in combined pathology cases, likely due to addressing both the angle-closure and trabecular outflow components simultaneously. Early lens extraction demonstrated particularly favorable outcomes in the combined group, with 72.4% achieving sustained IOP control without medication compared to 41.7% with medical/laser management alone.

#### Prognostic prediction model development

Based on comprehensive longitudinal data analysis, we developed and validated a prognostic prediction model specifically designed for combined XFS-PACG cases. The model incorporates eight key parameters: age, disease duration before diagnosis, baseline IOP, IOP fluctuation amplitude, angle configuration, presence of zonular instability, baseline visual field mean deviation, and lens status. Internal validation demonstrated robust predictive performance with area under the receiver operating characteristic curve (AUC) of 0.837 (95% CI 0.762–0.893) for predicting rapid progression (MD decline > 2dB/year).

The model stratifies patients into three risk categories (low, moderate, high) with corresponding 3-year rapid progression probabilities of 18.3%, 42.6%, and 76.8%, respectively. Risk stratification using this model effectively identified patients benefiting most from early aggressive intervention, with number needed to treat (NNT) of 3.2 in the high-risk group versus 12.7 in the low-risk group to prevent one case of rapid progression. Implementation of this prediction algorithm in clinical decision support systems could facilitate individualized treatment planning, optimizing resource allocation while minimizing vision loss in this challenging patient population. Importantly, the model’s distinctive weighting of factors specific to combined pathology resulted in significantly improved predictive accuracy compared to existing models developed for either isolated condition (AUC improvement 0.147 versus XFS-specific model and 0.168 versus PACG-specific model).

## Analysis of intervention effects and influencing factors

### Evaluation of pharmacological intervention effects

#### Comparative analysis of IOP-lowering efficacy

Comprehensive analysis of pharmacological intervention outcomes revealed distinctive response patterns in combined XFS-PACG patients compared to those with isolated pathologies. Prostaglandin analogues (PGAs), the established first-line therapy for open-angle glaucoma, demonstrated notably diminished efficacy in the combined pathology group, achieving mean IOP reduction of 22.7 ± 4.3% compared to 31.4 ± 5.8% in the XFS group and 26.1 ± 4.9% in the PACG group. This attenuated response appears related to the unique pathophysiological mechanisms in combined cases, where conventional uveoscleral outflow enhancement by PGAs fails to adequately address the complex outflow impediments. Beta-blockers similarly showed reduced effectiveness in the combined group (IOP reduction 18.3 ± 3.9%) compared to isolated XFS (23.7 ± 4.2%) and PACG (21.8 ± 4.0%) groups, likely reflecting the limited benefit of aqueous suppression alone in the setting of severe outflow compromise.

Interestingly, alpha-2 adrenergic agonists demonstrated comparable efficacy across all three groups (19.1 ± 3.8% in combined, 20.3 ± 4.1% in XFS, and 18.7 ± 3.7% in PACG), suggesting their dual mechanism of decreasing aqueous production while enhancing uveoscleral outflow may provide advantages in combined pathology. Carbonic anhydrase inhibitors (CAIs) showed modest efficacy across all groups but were particularly prone to tachyphylaxis in the combined group, with IOP-lowering effect declining from 20.6 ± 3.7% at 1 month to 13.2 ± 4.6% at 6 months, compared to more stable responses in XFS (18.9 ± 3.5% to 16.8 ± 3.8%) and PACG (19.4 ± 3.6% to 17.1 ± 3.9%) groups. The comparative efficacy of various pharmacological interventions across all diagnostic groups is detailed in Table 4.

**Table 4:** Comparative analysis of Pharmacological intervention effects.

Medication cass	Parameter	XFS group	PACG group	Combined XFS-PACG	Fixed combination therapy*	*p*-value
Prostaglandin analogues	IOP reduction (%)^†^	31.4 ± 5.8	26.1 ± 4.9	22.7 ± 4.3	28.6 ± 5.1	< 0.001
	Target IOP achieved (%)^‡^	56.8	47.2	32.4	53.3	< 0.001
	VF stability rate (%)^‡^	72.3	68.5	51.4	64.8	0.003
	Compliance score (1–10)^†^	8.2 ± 1.1	8.3 ± 1.0	8.1 ± 1.2	8.7 ± 0.9	0.072
	Adverse events (%)^‡^	21.2	23.6	27.6	25.7	0.038
Beta-blockers	IOP reduction (%)^†^	23.7 ± 4.2	21.8 ± 4.0	18.3 ± 3.9	25.4 ± 4.3	0.012
	Target IOP achieved (%)^‡^	41.5	38.6	26.7	44.8	0.007
	VF stability rate (%)^‡^	65.3	62.2	47.6	60.0	0.006
	Compliance score (1–10)^†^	7.4 ± 1.3	7.5 ± 1.2	7.3 ± 1.4	8.1 ± 1.0	0.064
	Adverse events (%)^‡^	28.0	26.8	33.3	30.5	0.041
Alpha-2 agonists	IOP reduction (%)^†^	20.3 ± 4.1	18.7 ± 3.7	19.1 ± 3.8	23.9 ± 4.2	0.083
	Target IOP achieved (%)^‡^	33.9	31.5	28.6	40.9	0.029
	VF stability rate (%)^‡^	61.9	59.8	45.7	57.1	0.011
	Compliance score (1–10)^†^	6.7 ± 1.5	6.8 ± 1.4	6.5 ± 1.6	7.8 ± 1.1	0.038
	Adverse events (%)^‡^	35.6	34.6	41.9	37.1	0.023
CAIs	IOP reduction (%)^†^	18.9 ± 3.5	19.4 ± 3.6	16.4 ± 3.5	22.7 ± 3.9	0.017
	Target IOP achieved (%)^‡^	28.8	30.7	21.9	38.1	0.006
	VF stability rate (%)^‡^	58.5	57.5	42.9	53.3	0.010
	Compliance score (1–10)^†^	7.0 ± 1.4	7.1 ± 1.3	6.8 ± 1.5	7.6 ± 1.2	0.054
	Adverse events (%)^‡^	31.4	30.7	38.1	34.3	0.031
Cost-effectiveness ratio	Cost per 1mmHg IOP reduction ($/mmHg)^†^	12.7	13.9	17.8	11.3	< 0.001

*Fixed combination therapy refers to prostaglandin/beta-blocker combination for XFS and combined XFS-PACG groups, and beta-blocker/CAI combination for PACG group. ^†^Analyzed using one-way ANOVA with Bonferroni post-hoc test ^‡^Analyzed using Chi-square test CAIs: Carbonic Anhydrase Inhibitors; IOP: Intraocular Pressure; VF: Visual Field.

#### Analysis of combination therapy efficacy

Given the suboptimal response to monotherapy, combination approaches were extensively evaluated, revealing significant differences in optimal regimens between groups. In the combined pathology group, the most effective dual therapy was prostaglandin analogue plus alpha-2 agonist (IOP reduction 28.6 ± 5.1%), significantly outperforming prostaglandin plus beta-blocker (25.4 ± 4.3%) and prostaglandin plus CAI (24.7 ± 4.5%) combinations. This contrasted with the XFS group, where prostaglandin plus beta-blocker yielded superior results (35.8 ± 6.1%), and the PACG group, where prostaglandin plus CAI was most effective (30.3 ± 5.3%).

Target IOP achievement rates (defined as IOP ≤ 18 mmHg with ≤ 20% fluctuation) further highlighted the therapeutic challenges in combined pathology, with only 32.4% of patients achieving targets on PGA monotherapy, compared to 56.8% in XFS and 47.2% in PACG groups. Triple therapy was required in 63.8% of combined pathology patients to achieve target IOP, compared to 37.3% in XFS and 42.5% in PACG groups. This differential response pattern necessitates consideration of a tailored pharmaco-therapeutic approach for the combined pathology population rather than extrapolating from management protocols developed for either isolated condition.

#### Adverse events and management strategies

Adverse event analysis revealed not only higher overall incidence in the combined pathology group (27.6% with PGAs, 33.3% with beta-blockers, 41.9% with alpha-2 agonists, and 38.1% with CAIs) but also distinctive patterns of reactions. Exacerbation of dry eye symptoms was particularly prevalent in the combined group (38.1% versus 22.9% in XFS and 26.8% in PACG), likely reflecting synergistic ocular surface disruption from both exfoliation material deposition and chronic angle-closure induced inflammation. Paradoxical IOP spikes following alpha-2 agonist discontinuation were significantly more common in the combined group (17.1% versus 7.6% in XFS and 6.3% in PACG), necessitating more gradual tapering protocols.

To optimize medication selection incorporating these multifaceted considerations, we developed and validated a Drug Selection Priority Index (DSI):

$$\:DSI=\left(\text{Efficacy\:coefficient}\times\:0.4\right)+\left(\text{Tolerability}\times\:0.3\right)+\left(\text{Compliance}\times\:0.2\right)+\left(\text{Cost}\times\:0.1\right)$$

When applied to the combined pathology population, this model yielded significantly different medication prioritization compared to either isolated condition, with prostaglandin/alpha-2 agonist fixed combinations achieving the highest DSI score (0.76) compared to prostaglandin/beta-blocker combinations (0.71) and prostaglandin monotherapy (0.68). Implementation of this tailored medication selection algorithm in a prospective validation cohort demonstrated significant improvements in 12-month outcomes, with 18.3% higher target IOP achievement rates compared to conventional stepwise therapy, highlighting the importance of condition-specific rather than generic pharmaceutical management approaches for this unique patient population.

### Analysis of laser intervention effects

#### Comparative analysis of laser treatment indications and efficacy

Comprehensive evaluation of laser interventions revealed distinct efficacy profiles across the three diagnostic groups, with particularly nuanced responses in the combined XFS-PACG cohort. Laser peripheral iridotomy (LPI), the established first-line intervention for angle-closure mechanisms, demonstrated significantly lower success rates in the combined pathology group compared to isolated PACG. Complete resolution of appositional angle closure following LPI was achieved in only 38.1% of combined cases compared to 72.4% in the PACG group, despite technically patent iridotomies confirmed by gonioscopy and anterior segment OCT. This diminished efficacy appears attributable to the unique anterior segment dynamics in combined pathology, where exfoliation material-induced iris rigidity and zonular weakness contribute to persistent angle crowding despite elimination of pupillary block.

Argon laser peripheral iridoplasty (ALPI), when applied as a secondary intervention for persistent appositional closure following LPI, demonstrated superior outcomes in the combined pathology group, with additional angle opening of 8.7 ± 2.3 degrees compared to 5.4 ± 1.8 degrees in the PACG group. This enhanced response likely reflects the particular benefit of mechanical iris reconfiguration in counteracting the exfoliation-induced iris rigidity characteristic of combined cases. Selective laser trabeculoplasty (SLT), typically reserved for open-angle mechanisms, showed surprisingly robust efficacy in combined pathology patients once angle-closure components were addressed, achieving mean IOP reduction of 6.9 ± 2.4 mmHg (26.3%) compared to 7.3 ± 2.6 mmHg (28.1%) in isolated XFS cases. This finding suggests the persistent importance of trabecular outflow dysfunction even after resolution of angle-closure mechanisms in combined pathology patients. Table 5 summarizes the comparative analysis of different laser intervention effects across all diagnostic groups.

**Table 5:** Comparative analysis of laser intervention effects.

Laser procedure	Parameter	XFS group	PACG group	Combined XFS-PACG	*p*-value
Laser peripheral iridotomy	IOP control rate (%)*^†^	N/A	72.4	38.1	< 0.001
	Angle opening (degrees)^‡^	N/A	12.7 ± 3.6	7.4 ± 2.8	< 0.001
	Adjunctive medication required (%)^†^	N/A	43.3	78.6	< 0.001
	Complication rate (%)^†^	N/A	8.7	17.1	0.012
	Retreatment rate (%)^†^	N/A	10.2	38.1	< 0.001
Argon laser peripheral iridoplasty	IOP control rate (%)*^†^	N/A	64.8	53.3	0.027
	Angle opening (degrees)^‡^	N/A	5.4 ± 1.8	8.7 ± 2.3	0.008
	Adjunctive medication required (%)^†^	N/A	57.1	66.7	0.042
	Complication rate (%)^†^	N/A	13.4	21.9	0.019
	Retreatment rate (%)^†^	N/A	23.6	33.3	0.031
Selective laser trabeculoplasty	IOP control rate (%)*^†^	64.4	N/A	53.3	0.023
	Mean IOP reduction (mmHg)^‡^	7.3 ± 2.6	N/A	6.9 ± 2.4	0.068
	Adjunctive medication required (%)^†^	47.5	N/A	61.9	0.013
	Complication rate (%)^†^	7.6	N/A	14.3	0.021
	Retreatment rate (%)^†^	32.2	N/A	44.8	0.017

*IOP control rate defined as IOP ≤ 18 mmHg with ≤ 20% fluctuation without medication augmentation . ^†^Analyzed using Chi-square test. ^‡^Analyzed using Student’s t-test N/A: Not Applicable; XFS: Exfoliation Syndrome; PACG: Primary Angle-Closure Glaucoma; IOP: Intraocular Pressure.

#### Post-laser anatomical and pressure dynamics

Anterior segment analysis following laser intervention revealed distinctive anatomical responses in the combined pathology group. Sequential imaging demonstrated that while LPI effectively eliminated the pupillary block component, 61.9% of combined cases exhibited persistent angle narrowing due to anterior rotation of the ciliary body and forward displacement of the lens-iris diaphragm—structural alterations attributed to progressive zonular weakness characteristic of exfoliation syndrome. Ultrasound biomicroscopy documented a mean anterior lens shift of 0.27 ± 0.08 mm over the 12-month post-LPI period in combined cases, compared to 0.09 ± 0.03 mm in the PACG group, directly correlating with progressive angle narrowing and IOP elevation despite initially successful intervention.

Pressure dynamics analysis revealed a distinctive pattern of transient post-laser IOP improvement followed by gradual deterioration in the combined group. Following LPI, 73.3% of combined pathology patients demonstrated initial IOP reduction (mean 5.8 ± 2.1 mmHg), but this benefit diminished progressively, with 62.9% returning to within 20% of pre-laser IOP levels by 12 months. This contrasted with more sustained pressure control in the PACG group, where only 27.6% experienced similar deterioration. Diurnal IOP studies further revealed that while morning pressure peaks (characteristic of pupillary block) were effectively eliminated by LPI in both groups, the combined pathology group maintained the afternoon pressure elevations typical of exfoliation syndrome, highlighting the multifactorial pressure dynamics requiring comprehensive management approaches.

#### Long-term durability and safety profile

Longitudinal follow-up demonstrated significant differences in laser intervention durability across diagnostic groups. Kaplan-Meier survival analysis (defining failure as IOP > 21 mmHg or > 20% above target on two consecutive visits) revealed median LPI survival time of 14.3 months in the combined group compared to 31.7 months in the PACG group. Similarly, SLT demonstrated reduced longevity in combined cases (median 16.9 months) versus isolated XFS (22.7 months). ALPI exhibited the most durable effect in the combined pathology group among laser modalities, with median efficacy duration of 20.3 months, likely reflecting its mechanical reconfiguration of anterior segment architecture rather than reliance on physiological outflow pathways progressively compromised by exfoliation material.

Safety profile assessment revealed higher complication rates across all laser modalities in the combined pathology group. Post-LPI complications were particularly prevalent, with transient IOP spikes (> 30% above baseline) occurring in 21.9% of combined cases versus 9.4% in PACG cases, despite identical pre-treatment protocols. Persistent inflammation (> 2 + anterior chamber cells at 1 week) occurred in 14.3% of combined cases versus 5.5% of PACG cases, likely reflecting altered blood-aqueous barrier function in exfoliation syndrome. Post-SLT complications similarly showed higher incidence in combined cases, with substantial pigment dispersion occurring in 19.0% versus 8.5% in isolated XFS, necessitating modified treatment parameters (reduced energy and treatment spots) in this population to maintain acceptable safety profiles.

#### Optimization of combined laser-pharmacological approaches

The distinctive response patterns of combined pathology patients necessitated development of tailored combination approaches to optimize outcomes. Sequential intervention analysis demonstrated that combined pathology patients derived greatest benefit from a staged approach: initial LPI followed by ALPI at 2–4 weeks (if angle remained occludable), and subsequent SLT at 6–8 weeks (if IOP remained elevated despite open angles). This comprehensive sequential approach achieved target IOP in 64.8% of combined cases at 12 months, significantly outperforming either isolated laser modality (38.1% for LPI alone, 53.3% for ALPI alone, 53.3% for SLT alone).

Pharmacological augmentation strategies revealed that alpha-2 agonists provided optimal adjunctive therapy following laser intervention in combined cases, with 72.4% achieving target IOP when added to laser therapy, compared to 61.9% with prostaglandin analogues and 57.1% with beta-blockers. This contrasted with isolated PACG, where prostaglandins demonstrated superior adjunctive efficacy (76.4%), and isolated XFS, where beta-blockers showed greatest synergy with SLT (69.5%). The unique efficacy of alpha-2 agonists in the post-laser combined pathology setting potentially reflects their complementary mechanisms addressing both aqueous production and uveoscleral outflow pathways—particularly valuable given the complex outflow impedance characteristic of this condition.

Medication requirement analysis further demonstrated that successful sequential laser therapy reduced mean medication burden in combined pathology patients from 2.7 ± 0.8 to 1.4 ± 0.6 agents, representing significant improvement in treatment burden despite the challenging nature of these cases. This finding supports the role of comprehensive laser intervention as a medication-sparing approach even in cases with complex underlying pathophysiology, potentially enhancing long-term adherence and minimizing cumulative medication side effects in this predominantly elderly population.

### Surgical intervention effects and optimal intervention timing

#### Comparative analysis of surgical approaches

Comprehensive evaluation of surgical interventions revealed significant efficacy differences across the three diagnostic groups, with particularly distinctive outcomes in the combined XFS-PACG cohort. Trabeculectomy, while effective in all groups, demonstrated lower complete success rates (defined as IOP ≤ 18 mmHg without medications) in the combined pathology group (53.3%) compared to isolated XFS (67.8%) and PACG (69.3%) groups at 24 months postoperatively. This reduced efficacy appears attributable to the accelerated subconjunctival fibrosis observed in combined cases, with ultrasound biomicroscopy revealing more pronounced bleb wall thickening (mean 0.42 ± 0.11 mm) compared to XFS (0.31 ± 0.09 mm) and PACG (0.28 ± 0.08 mm) groups by 12 months, despite identical mitomycin-C application protocols.

Glaucoma drainage device (GDD) implantation demonstrated more comparable efficacy across groups, with complete success rates of 64.8% in combined pathology compared to 68.6% in XFS and 70.1% in PACG at 24 months. The relative preservation of GDD efficacy in combined cases likely reflects the device’s mechanism of bypassing both trabecular and subconjunctival outflow pathways compromised by exfoliation material. Combined phacoemulsification with intraocular lens implantation and minimally invasive glaucoma surgery (Phaco-MIGS) emerged as particularly effective in the combined pathology group, achieving complete success in 72.4% of cases compared to 58.5% in XFS and 66.9% in PACG groups. This superior outcome appears related to simultaneous addressing of multiple pathogenic mechanisms including pupillary block, lens-induced angle crowding, and trabecular outflow dysfunction—all significant contributors in combined pathology. A comprehensive analysis of surgical outcomes and complications across different intervention approaches and diagnostic groups is presented in Table 6.

**Table 6:** Analysis of surgical outcomes and complications.

Surgical procedure	Parameter	Diagnostic group			Intervention timing	
		XFS group	PACG group	Combined XFS-PACG	Early surgery*	Delayed surgery^†^
Trabeculectomy	IOP control rate (%)^‡§^	67.8	69.3	53.3	62.9	43.8
	Medication reduction (%)§	88.1	83.5	76.2	85.7	66.7
	Visual acuity improvement^¶^	0.07 ± 0.12	0.09 ± 0.14	0.05 ± 0.13	0.11 ± 0.16	-0.03 ± 0.15
	Complication rate (%)§	21.2	23.6	32.4	24.8	40.0
	Reoperation rate (%)§	15.3	16.5	26.7	19.0	34.3
Glaucoma drainage device	IOP control rate (%)^‡§^	68.6	70.1	64.8	71.4	57.1
	Medication reduction (%)§	76.3	78.0	71.4	77.1	65.7
	Visual acuity improvement^¶^	0.05 ± 0.11	0.08 ± 0.13	0.02 ± 0.14	0.08 ± 0.15	-0.05 ± 0.17
	Complication rate (%)§	30.5	26.0	36.2	28.6	44.8
	Reoperation rate (%)§	13.6	11.8	17.1	12.4	22.9
Phaco-combined surgery	IOP control rate (%)^‡§^	58.5	66.9	72.4	81.0	63.8
	Medication reduction (%)§	72.0	81.9	85.7	90.5	81.0
	Visual acuity improvement^¶^	0.31 ± 0.18	0.36 ± 0.19	0.28 ± 0.17	0.35 ± 0.18	0.21 ± 0.19
	Complication rate (%)§	18.6	22.8	31.4	23.8	39.0
	Reoperation rate (%)§	11.0	9.4	14.3	9.5	19.0
Specific complications (%)^§^						
Shallow anterior chamber		11.9	14.2	19.0	13.3	24.8
Choroidal detachment		8.5	10.2	15.2	10.5	20.0
Malignant glaucoma		2.5	6.3	9.5	4.8	14.3
Retinal detachment		1.7	0.8	4.8	1.9	7.6

*Early surgery defined as surgical intervention within 6 months of diagnosis or after failure of first-line therapy. ^†^Delayed surgery defined as intervention after ≥ 18 months of maximal medical therapy or after failure of multiple laser interventions. ^‡^IOP control rate defined as IOP ≤ 18 mmHg with ≤ 20% fluctuation without medication at 24 months. ^§^Analyzed using Chi-square test, *p* < 0.001 for group comparisons. ^¶^Visual acuity improvement reported as mean logMAR change from baseline at 12 months (positive values indicate improvement), analyzed using one-way ANOVA, *p* < 0.001 XFS: Exfoliation Syndrome; PACG: Primary Angle-Closure Glaucoma; IOP: Intraocular Pressure.

#### Postoperative complications and management strategies

Postoperative complication analysis revealed distinctive patterns in the combined pathology group, with significantly higher overall complication rates across all surgical modalities. Following trabeculectomy, combined pathology patients demonstrated higher incidence of early hypotony (28.6% versus 16.9% in XFS and 15.7% in PACG), prolonged inflammation (23.8% versus 13.6% in XFS and 11.8% in PACG), and bleb encapsulation (31.4% versus 19.5% in XFS and 18.1% in PACG). Notably, complications characteristic of both underlying conditions were observed, including exfoliation-related zonular instability manifesting as progressive anterior chamber shallowing and PACG-related malignant glaucoma (9.5% incidence compared to 2.5% in XFS and 6.3% in PACG).

Prevention and management strategies optimized for the combined pathology population included modified surgical techniques with tighter scleral flap suturing to mitigate early hypotony risk, extended postoperative anti-inflammatory regimens (topical steroids tapered over 12 weeks versus standard 6–8 weeks), and prophylactic peripheral iridoplasty to reduce malignant glaucoma risk. For combined phacoemulsification procedures, capsular tension rings were employed routinely in combined pathology cases regardless of intraoperative zonular dialysis observation, reducing late IOL dislocation rates from 11.4 to 3.8% in our series. Additional management modifications included earlier intervention with needling procedures for encapsulating blebs (initiated at 4 weeks postoperatively versus traditional 8–12 weeks) and more aggressive aqueous suppressant use in the immediate postoperative period to reduce fibroblast activation.

#### Influence of surgical timing on outcomes

Timing of surgical intervention emerged as a critical determinant of outcomes, particularly in the combined pathology group. Stratification by intervention timing revealed significantly better outcomes with early surgical intervention (defined as surgery within 6 months of diagnosis or after failure of first-line therapy) compared to delayed intervention (after ≥ 18 months of maximal medical therapy or multiple laser attempts). Early surgery in combined pathology achieved complete success rates of 62.9% with trabeculectomy, 71.4% with GDD, and 81.0% with Phaco-MIGS, compared to 43.8%, 57.1%, and 63.8% respectively with delayed intervention.

Multivariate analysis identified duration of preoperative medical therapy as an independent negative predictor of surgical success in combined pathology (OR 0.78 per year, 95% CI 0.67–0.91), with each additional year of preoperative medical therapy associated with 22% reduced likelihood of complete success. This timing effect was less pronounced in isolated XFS (OR 0.91 per year) and PACG (OR 0.89 per year) groups. This differential impact of delayed intervention likely reflects the accelerated fibrotic response and progressive zonular compromise characteristic of combined pathology, with chronic inflammation from prolonged medical therapy further exacerbating these processes.

#### Optimized stepped intervention strategy

Based on comprehensive outcome analysis, we developed an optimized stepped intervention protocol specifically for combined XFS-PACG patients. The protocol advocates early lens extraction (phacoemulsification with IOL) combined with a MIGS procedure (typically ab-interno trabeculotomy or trabecular micro-bypass stent) as first-line surgical approach in patients with visually significant cataract or clear lens with anterior chamber depth < 2.3 mm. For cases with deeper anterior chambers or following previous lens extraction, early trabeculectomy with tight flap suturing and extended antimetabolite application (mitomycin-C 0.4 mg/mL for 3 min) is recommended as primary intervention.

For cases failing primary surgery or demonstrating risk factors for trabeculectomy failure (e.g., previous ocular surgery, inflammatory conditions), GDD implantation is recommended, with preference for larger plate devices (Baerveldt 350 mm^2^ rather than smaller Ahmed valves) to enhance long-term pressure control. This tailored approach achieved 24-month complete success rates of 77.1% in a prospective validation cohort, representing significant improvement over historical rates (53.3–64.8%) with non-optimized approaches in comparable combined pathology populations.

#### Individualized intervention decision support model

To facilitate personalized surgical decision-making, we developed and validated a predictive algorithm incorporating 12 parameters identified through multivariate analysis as significant predictors of surgical outcomes in combined pathology. Key variables include age, angle configuration, lens status, zonular integrity assessment, baseline IOP, number and duration of preoperative medications, previous laser interventions, and corneal endothelial cell density. The resulting nomogram stratifies patients into risk categories with corresponding surgical approach recommendations, achieving 81.3% accuracy in predicting successful outcomes in the validation cohort.

Implementation of this decision support tool in a prospective pilot study demonstrated significant improvements in surgical success rates (83.8% versus 67.1% with conventional decision-making) and reduction in complication rates (19.0% versus 31.4%), highlighting the value of condition-specific algorithmic approaches to surgical planning. Integration of this prediction model into electronic health record systems could potentially standardize management approaches while accommodating individual patient factors, optimizing outcomes in this challenging patient population characterized by complex pathophysiology and elevated surgical risk profile.

## Conclusion

This comprehensive investigation of combined exfoliation syndrome and angle-closure glaucoma has identified distinctive clinicopathological characteristics that differentiate this entity from either isolated condition. The combined pathology demonstrates unique features including bimodal diurnal IOP fluctuations, asymmetric angle closure correlating with exfoliation material distribution, compromised pupillary dynamics, and accelerated zonular weakness with progressive anterior lens displacement. Disease progression analysis revealed significantly more aggressive natural history in combined cases, with visual field deterioration rates approximately twice those observed in isolated pathologies (-2.9 dB/year versus − 1.7 and − 1.4 dB/year in XFS and PACG respectively), highlighting the critical importance of timely diagnosis and intervention.

Therapeutic response evaluation demonstrated that combined XFS-PACG cases require distinctive management approaches rather than protocols extrapolated from either isolated condition. Pharmacologically, prostaglandin-alpha2agonist combinations proved most effective despite conventional prostaglandin-betablocker combinations being optimal in isolated XFS. Laser interventions showed limited durability in combined pathology, with LPI alone achieving sustained control in only 38.1% of cases, necessitating sequential multimodal laser approaches. Surgically, early lens extraction combined with MIGS procedures demonstrated superior outcomes (72.4% complete success) compared to traditional filtering surgery (53.3%) in combined cases, contrasting with effectiveness patterns in isolated pathologies.

The optimal management pathway for combined XFS-PACG involves early diagnosis facilitated by recognition of pathognomonic clinical features, prompt intervention with combined phacoemulsification-MIGS as first-line surgical approach when appropriate, and close monitoring with anticipatory management of characteristic complications. The individualized intervention decision support algorithm developed through this research demonstrates potential to significantly improve outcomes through condition-specific risk stratification and surgical planning.

Study limitations include the relatively short follow-up duration (36 months) compared to the chronic disease course and the need for external validation of prediction models in diverse populations. Future research should focus on molecular mechanisms underlying the synergistic pathophysiology, longitudinal evaluation of novel interventional techniques, and development of preventive strategies targeting the accelerated fibrotic response characteristic of this challenging clinical entity.

## Data Availability

The datasets used and/or analysed during the current study available from the corresponding author on reasonable request.
